# Drug‐Induced Vitiligo: FDA Adverse Events Reporting System (FAERS) and Canada Vigilance Adverse Reaction (CAVR) Database Analysis and Literature Review

**DOI:** 10.1111/jocd.71149

**Published:** 2026-08-24

**Authors:** Yuhao Li, Yi Ou, Luyue Chang, Ruiyao Wang, Xinyi Shao, Jingbo Zhang, Jin Chen

**Affiliations:** ^1^ Department of Dermatology The First Affiliated Hospital of Chongqing Medical University Chongqing China

**Keywords:** adverse drug reaction, Canada vigilance adverse reaction (CAVR) database, FDA adverse events reporting system (FAERS), pharmacovigilance, vitiligo

## Abstract

**Background:**

Vitiligo is a common chronic acquired pigmentation disease, that usually occurs with an asymptomatic whitish patch or macule. Although previous studies have reported several cases of drug‐induced vitiligo, there are few comprehensive studies determining the drugs that induce vitiligo.

**Aims:**

This study investigated the risk factors for drug‐induced vitiligo by analyzing a large dataset from the FDA Adverse Event Reporting System (FAERS) and the Canada Vigilance Adverse Reaction (CVAR) database.

**Methods:**

Disproportionality analysis using the reporting odds ratio (ROR) method, with *p*‐values adjusted via Bonferroni correction, identified significant signals. Drugs with positive signals were analyzed for annual reporting trends using linear regression. Time‐to‐onset analysis was also performed.

**Results:**

We identified 1910 reports in FAERS and 316 reports in the CAVR database related to vitiligo. Disproportionality analysis revealed significant signals for 57 drugs in FAERS and 7 drugs in the CAVR database. Most were antineoplastic drugs and immune inhibitors with significant annual increases in vitiligo reports. Through reviewing reports of immune inhibitors induced vitiligo, we found that the severity of the disease may be related to the occurrence of vitiligo AE. The median time to onset of vitiligo was 86 days, with over half of the cases occurring within 90 days of initiating medication.

**Conclusions:**

This study confirmed safety signals associated with various antineoplastic agents and immune inhibitors, highlighting the importance of ongoing pharmacovigilance. Further mechanistic investigations are needed to clarify the pathological pathways underlying drug‐induced vitiligo.

## 1 Introduction

Vitiligo is a common acquired pigmentary disease characterized by well‐demarcated areas of white macules. Affecting approximately 28.5 million individuals worldwide [1], it poses a substantial psychological burden and compromises quality of life due to its frequent involvement of visible and cosmetically sensitive areas. The pathogenesis of vitiligo is complex, and multiple factors have been proposed, such as genetic, oxidative stress, autoimmune, and autoinflammatory [2].

Notably, there has been increasing attention to case reports of drug‐induced vitiligo in recent years, including drugs such as imiquimod, biologic drugs, and interferons [3, 4]. Anthony et al. investigated drug‐induced vitiligo using the World Health Organization pharmacovigilance database, VigiBase [5], while Lu et al. summarized vitiligo cases associated with the use of type 2 immune inhibitors [6]. However, these studies primarily relied on single data sources and lacked systematic signal verification across multiple pharmacovigilance systems. In addition, the temporal characteristics of adverse events (AEs) related to drug‐induced vitiligo remain insufficiently explored.

The FDA adverse events reporting system (FAERS) and Canada vigilance adverse reaction (CAVR) database collect voluntarily submitted AE reports, which include patient characteristics, drug use, adverse reactions, and outcomes [7, 8]. The FAERS and CAVR databases were recently employed to identify AEs, such as anticancer therapy‐related dermatologic AEs [9] and drug‐induced glaucoma [10]. Therefore, we performed a comprehensive study to investigate drug‐induced vitiligo using the FAERS and CAVR databases. Additionally, we conducted an analysis of the onset timings of AEs associated with drug‐induced vitiligo to gain deeper insights into the temporal patterns of this condition. The objective of this research is to furnish a scientific foundation for drug safety monitoring and to provide clinical references that enhance medication safety for patients.

## 2 Materials and Methods

### 2.1 Data Sources, Management, and Study Design

FAERS contains millions of real‐world AE reports submitted by healthcare providers, patients, legal representatives, and pharmaceutical manufacturers, comprising seven dataset types: demographic and administrative information (DEMO), drug information (DRUG), adverse event coding (REAC), patient outcomes (OUTC), report sources (RPSR), therapy dates (THER), and drug indications (INDI). Each report classifies a drug's role in an AE as primary suspect (PS), secondary suspect (SS), interacting (I), or concomitant (C) [11]. Managed by Health Canada, the CVAR database has documented post‐market adverse reactions in Canada since 1965, which are voluntarily submitted by consumers and health professionals and mandatorily submitted by manufacturers and distributors, include patient demographics, medication use, adverse reactions, and outcomes.

This research initially employs the FAERS database spanning from 2004 through the fourth quarter of 2024 and the CVAR database from 1990 through 2024 to retrieve AE reports associated with vitiligo. To address potential duplicate reports from diverse FDA data submissions, we followed FDA guidelines: when CASEID was identical, the most recent FDA_DT and highest PRIMARYID were retained. All reported AEs were systematically coded using the Medical Dictionary for Regulatory Activities (MedDRA) classification system. MedDRA's hierarchical framework consists of five levels: System Organ Class (SOC), High‐Level Group Term (HLGT), High‐Level Term (HLT), Preferred Term (PT), and Lowest Level Term (LLT) [12]. In the present study, we retrieved all AE reports containing the PT “vitiligo” and prioritized drugs categorized as “PS.”

### 2.2 Statistical Analysis

#### 2.2.1 Disproportionality Analysis

This research adopted time‐series plots and linear regression analysis to assess the annual reporting trends of drug‐related categories with positive signals, while adjusting *p*‐values through the Bonferroni method. Disproportionality analysis, a widely used approach in pharmacovigilance, relied on the classical 2 × 2 contingency table (Table 1) to analyze the occurrence frequencies of target drugs and target AEs in comparison with background frequencies, thereby establishing statistical correlations between drugs and AEs. The reporting odds ratio (ROR) algorithm was utilized to identify drug‐related AE signals. The calculation of the ROR and its 95% CI was performed as follows:

$$\mathrm{ROR}=\frac{\mathrm{ad}}{\mathrm{bc}}\;95\%\mathrm{CI}=e^{\ln\left(\mathrm{ROR}\right)\pm 1.96\times \sqrt{\frac{1}{a}+\frac{1}{b}+\frac{1}{c}+\frac{1}{d}}}$$

**TABLE 1:** 2 × 2 contingency table for disproportionality analysis.

	Target AEs	All other AEs
Target drug	a	b
All other drugs	c	d

*Note:* “a” represents the number of specific AEs, related to the target drug combination, “b” represents the number of other AEs related to the target drug, “c” represents the number of AEs related to other drugs involving the target AE, and “d” represents the number of other AEs unrelated to the target drug.

Abbreviation: AEs, adverse events.

A positive signal for AEs was identified under the following criteria: the lower bound of the 95% CI for the ROR exceeded 1.0, and the target AE had at least three reported cases (a ≥ 3). The ROR value also functioned as a metric for comparing AE risks across different drugs, with a higher ROR indicating a greater likelihood of drug‐induced vitiligo [13]. All statistical analyses were executed using R software version 4.4.3. Statistical analyses were two‐sided, and *p* < 0.05 was considered statistically significant.

#### 2.2.2 Sensitivity Analysis for Indication‐Related Confounding

To assess the potential influence of confounding by indication, additional sensitivity analyses were performed for drugs with substantial overlap between their therapeutic indications and diseases associated with an increased risk of vitiligo. For immune checkpoint inhibitors, including nivolumab, pembrolizumab, and atezolizumab, melanoma‐related reports were identified based on reported indication terms (INDI_PT) containing “melanoma” and were excluded before repeating disproportionality analyses. For dupilumab, reports associated with atopic dermatitis‐related indications were excluded. For psoriasis‐associated biologic agents, including secukinumab, ixekizumab, risankizumab, guselkumab, and ustekinumab, psoriasis‐related reports were excluded. After exclusion of indication‐related reports, ROR values with corresponding 95% CIs were recalculated using the same criteria as the primary analysis.

#### 2.2.3 Time‐to‐Onset Analysis

The time‐to‐onset was defined as the duration between the therapy initiation date (START_DT in the THER file) and the event date (EVENT_DT in the DEMO file). Reports were excluded if they contained input errors (e.g., EVENT_DT preceding START_DT), inaccurate dates, or duplicate entries. In the present study, the median and quartiles were employed to characterize the time‐to‐onset of AEs.

## 3 Results

### 3.1 Baseline Characteristics of Vitiligo

**FIGURE 1:**
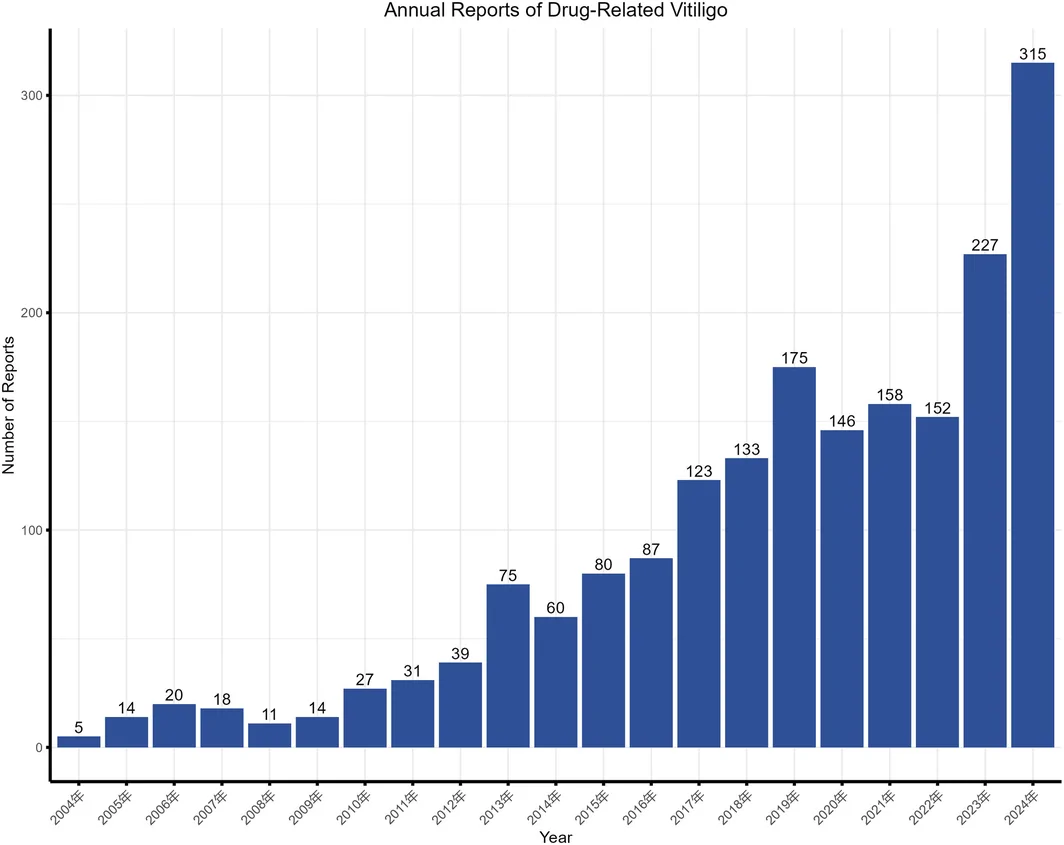
Annual reporting trends. The annual trend in the number of adverse event reports related to vitiligo from 2004 to the fourth quarter of 2024 in FAERS. FAERS, FDA Adverse Event Reporting System.

After initial screening, a total of 1910 reports in FAERS and 316 reports in the CAVR database were identified. Baseline characteristics of patients with drug‐related vitiligo are outlined in Table 2. In FAERS, reports of drug‐induced vitiligo exhibited a progressive upward trend (Figure 1; Table S1), with the peak annual reporting occurring in 2024 (315 cases, 16.49%). Among individuals with drug‐related vitiligo, females constituted 45.87% of cases, demonstrating a higher prevalence than males (35.86%). The median age of the patient cohort was 54.00 years (interquartile range [IQR]: 40.00–66.00), and the median weight was 72.30 kg. More than half (51.31%) of the reports were submitted by healthcare professionals. The United States accounted for the highest case burden (51.47%), with subsequent contributions from France (7.33%), Germany and the United Kingdom (each 4.40%), Canada (3.25%), and Brazil (2.67%). Detailed case reports from other nations are provided in Table S2. Similarly, we identified a median age of 53.00 years and 26.58% males in CAVR. Most of them were reported by healthcare providers.

**TABLE 2:** Basic characteristics of patients with drug‐related vitiligo from the FAERS database and CAVR database.

Characteristics	FAERS	CVAR
	Drug‐related vitiligo (*N* = 1910)	Drug‐related vitiligo (*N* = 316)
Age		
Median (IQR)	54 (40–66)	53 (45–59)
Unknown	1512 (79.16%)	14 (4.43%)
Gender		
Female	876 (45.87%)	230 (72.79%)
Male	685 (35.86%)	84 (26.58%)
Unknown	349 (18.27%)	2 (0.63%)
Weight		
Mean (SD)	72.3 (24.3)	72.5 (19.1)
Unknown	791 (41.41%)	208 (65.82%)
Occupation of reporter		
Physician	667 (34.92%)	24 (7.59%)
Pharmacist	63 (3.30%)	3 (0.95%)
Other health‐professional	250 (13.09%)	108 (34.18%)
Lawyer	17 (0.89%)	N/A
Consumer	575 (30.1%)	29 (9.18%)
Unknown	41 (2.15%)	152 (48.10%)
Country of reporter		
United States	983 (51.47%)	N/A
France	140 (7.33%)	N/A
Germany	84 (4.40%)	N/A
United Kingdom	84 (4.40%)	N/A
Canada	62 (3.25%)	N/A
Brazil	51 (2.67%)	N/A
Italy	47 (2.46%)	N/A
Country not specified	38 (2.00%)	N/A
Spain	35 (1.83%)	N/A
Japan	32 (1.68%)	N/A
Other	354 (18.53%)	N/A
Outcomes		
Death	43 (2.25%)	N/A
Life threatening	29 (1.52%)	N/A
Hospitalization	254 (13.29%)	3 (0.95%)
Disability	66 (3.46%)	2 (0.63%)
Required intervention	4 (0.21%)	N/A
Congenital anomaly	3 (0.16%)	N/A
Other serious	814 (42.62%)	273 (86.39%)
Not serious	N/A	38 (12.03%)
Unknown	697 (36.49%)	N/A

Abbreviations: CAVR, Canada vigilance adverse reaction; FAERS, FDA adverse events reporting system; N/A, not applicate.

### 3.2 Signal Detection and Validation

**FIGURE 2:**
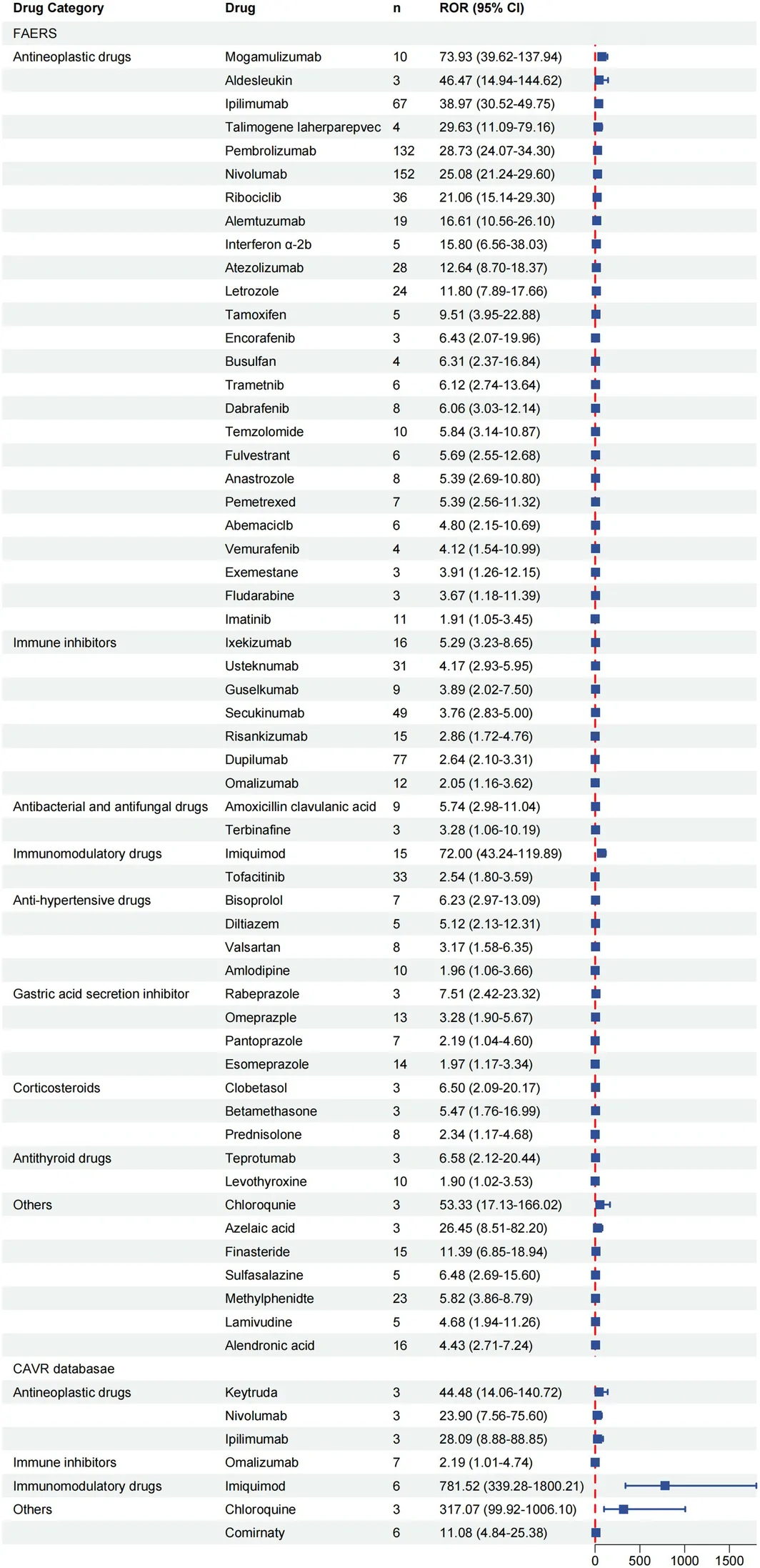
Forest plot of drugs associated with vitiligo in the FAERS and CAVR database. Forest plot of ROR analysis for 57 drugs with positive vitiligo signals in FAERS and 7 in CAVR database. CAVR, Canada vigilance adverse reaction; CI, confidence interval; FAERS, FDA Adverse Event Reporting System; ROR, reporting odds ratio.

The ROR method was implemented for detecting AE signals in drugs (Table S3). A total of 57 vitiligo‐related drugs were identified in FAERS (Figure 2). The drug categories included antineoplastic drugs (26/57), immune inhibitors (7/57), antibacterial and antifungal drugs (2/57), immunomodulatory drugs (2/57), antihypertensive drugs (4/57), gastric acid secretion inhibitors (4/57), corticosteroids (3/57), antithyroid drugs (2/57) and others (7/57). Agents associated with significantly higher reporting odds included imiquimod (ROR: 72.00; 95% CI: 43.24–119.89), chloroquine (ROR: 53.33; 95% CI: 17.13–166.02), mogamulizumab (ROR: 73.93; 95% CI: 39.62–137.94), aldesleukin (ROR: 46.47; 95% CI: 14.94–144.62) and ipilimumab (ROR: 38.97; 95% CI: 30.52–49.75). In the CAVR database, 7 drugs were identified with similarly high RORs, including imiquimod (ROR: 781.52; 95% CI: 339.28–1800.21) and chloroquine (ROR: 317.07; 95% CI: 99.92–1006.10).

To evaluate the potential influence of indication‐related confounding, indication‐based sensitivity analyses were conducted. After excluding melanoma‐related reports, the disproportionality signals remained significant for nivolumab and pembrolizumab, with RORs of 9.20 (95% CI, 6.64–12.75) and 8.04 (95% CI, 2.01–32.17), respectively. Similarly, exclusion of atopic dermatitis‐related reports for dupilumab did not attenuate the association (ROR, 3.17; 95% CI, 2.29–4.39). For psoriasis‐associated biologics, the signals remained generally consistent after excluding psoriasis‐related indications, although some estimates showed wider confidence intervals due to reduced sample sizes (Table S4).

### 3.3 Trend Analysis of Drug‐Related Vitiligo Incidents

**FIGURE 3:**
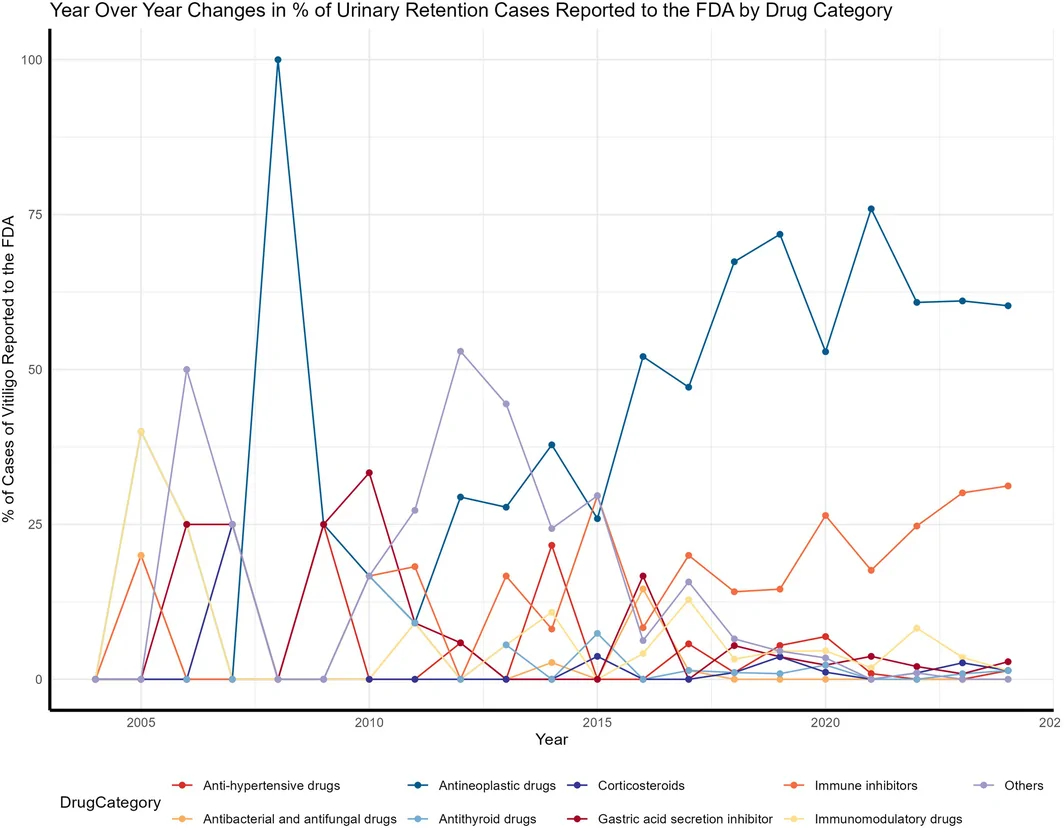
Annual time series plot. Changes in the percentage of vitiligo cases reported to the FAERS associated with various drug classes from 2004 to 2024. FAERS, FDA Adverse Event Reporting System.

This study examined vitiligo‐related positive signal drugs reported to the FDA. Figure 3 displays the temporal distribution of these reports. Furthermore, linear regression was performed for each primary vitiligo‐associated drug class (Table 3). Antineoplastic agents demonstrated an average annual increase of 2.50% (95% CI: 0.85–4.14, *p*‐adjust = 0.01), rising from 0.00% in 2000 to 60.28% in 2024, representing the highest growth rate among all drug categories. Similarly, immune inhibitors exhibited an average annual rise of 1.27% (95% CI: 0.68–1.87, *p*‐adjust < 0.001) in FDA reports. We also confirmed this upward trend of immune inhibitors (1.35%, 95% CI: 0.52–2.19, *p*‐adjust < 0.05) in the CAVR database. Other drug categories (antibacterial and antifungal drugs, immunomodulatory drugs, antihypertensive drugs, gastric acid secretion inhibitors, corticosteroids, and antithyroid drugs) showed stable trends over time (*p*‐adjust > 0.05).

**TABLE 3:** Linear regression analysis of the percentage of vitiligo cases associated with different drug classes. For each drug class, the slope, 95% CI, *p*‐adjust, and the percentages in 2004 and 2024 are included.

Drug category	% change per year (95% CI)	% in 2004	% in 2024	*p*	*p*‐adjust^a^
**FAERS**					
Antineoplastic drugs	2.50 (0.85,4.14)	0.00	60.28	0.004	**0.01**
Immune inhibitors	1.27 (0.68,1.87)	0.00	31.21	< 0.001	**< 0.001**
Antibacterial and antifungal drugs	−0.45 (−0.98,0.07)	0.00	0.00	0.087	0.173
Immunomodulatory drugs	−0.46 (−1.25,0.20)	0.00	1.42	0.194	0.389
Anti‐hypertensive drugs	−0.03 (−0.58,0.51)	0.00	1.42	0.898	1
Gastric acid secretion inhibitor	−0.66 (−1.41,0.09)	0.00	2.84	0.083	0.16
Corticosteroids	−0.12 (−0.54,0.29)	0.00	1.42	0.54	1
Antithyroid drugs	−0.06 (−0.38,0.27)	0.00	1.42	0.723	1
**CAVR**		**% in 1990**	**% in 2024**		
Antineoplastic drugs	0.37 (−0.20,0.93)	0.00	0.00	0.2	0.401
Immune inhibitors	1.35 (0.52,2.19)	0.00	84.62	0.002	**0.004**
Immunomodulatory drugs	−0.22 (−1.20,0.75)	0.00	0.00	0.644	1

*Note:* Bold values indicate statistically significant *p*‐adjust.

Abbreviations: CAVR, Canada vigilance adverse reaction; CI, confidence interval; FAERS, FDA adverse events reporting system.

^a^ *p*‐adjust were adjusted using the Bonferroni method.

### 3.4 Onset Time of Vitiligo

**FIGURE 4:**
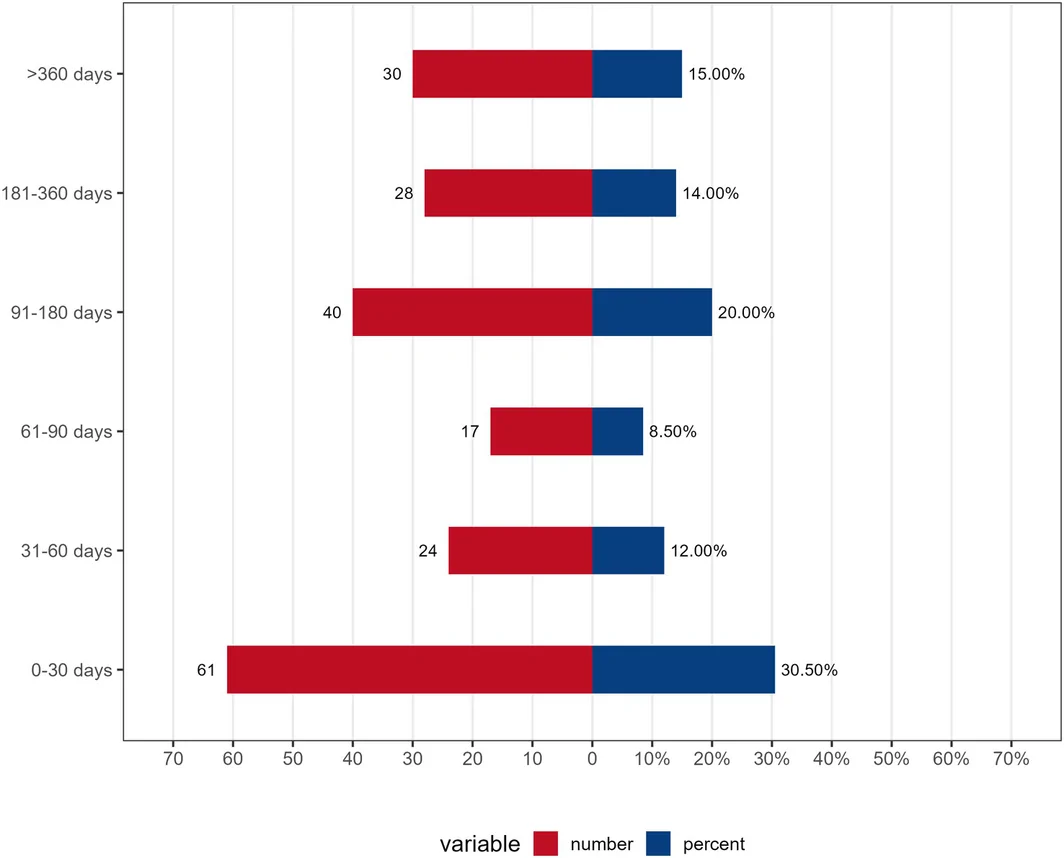
Time to onset of drug‐related vitiligo. The number and percent of time from the start of medication to the occurrence of vitiligo adverse reaction in FAERS. FAERS, FDA Adverse Event Reporting System.

Following the exclusion of duplicate and inaccurate reports, 200 reports provided the onset time data (Figure 4). The median time to vitiligo onset following drug exposure was 86 days (IQR: 21.00–212.25). Most cases of vitiligo occurred within 30 days of medication initiation (*n* = 61, 30.50%), but vitiligo could still occur over a year after starting the medication (*n* = 30, 15.00%).

## 4 Discussion

This study investigated reporting trends of major drug classes linked to vitiligo‐related AEs from 2004 to 2024 and employed statistical analyses to quantify temporal trends. Further signal detection identified 57 drugs in FAERS and 7 drugs in the CAVR database related to vitiligo, most of which were antineoplastic drugs and immune inhibitors. Of these, two drugs (imiquimod and chloroquine) were identified with similarly high RORs in both databases. However, most of these drugs do not contain vitiligo as a potential adverse effect in their labels, leading to an unawareness of their possible AE of vitiligo. Our study provided data support for the management of clinical medication safety.

Over the last 20 years, our analysis revealed a marked increase in drug‐induced vitiligo cases from 5 in 2004 to 315 in 2024. This upward trajectory could potentially stem from prompting disease awareness, expanded medication utilization, and more rigorous monitoring protocols for vitiligo [1]. Our findings demonstrated a greater prevalence among female patients compared with males, aligning with previous findings [14, 15]. This discrepancy may be linked to susceptibility to autoimmune diseases and greater attention to appearance in females. Furthermore, the data indicated that the highest reporting frequencies occurred in five developed nations. This observation likely reflects their advanced pharmacovigilance infrastructure, greater health awareness among both medical professionals and the general population, and more stringent regulatory standards for adverse event reporting.

Several studies have shown an association between the appearance of vitiligo and the response to treatment with immune checkpoint inhibitors (ICIs) in patients with melanoma [16, 17, 18]. The occurrence of vitiligo as an AE has also been observed with the use of immune inhibitors, especially with anti‐tumor necrosis factorα (TNFα) and anti‐interleukin‐17 (IL‐17) drugs [4]. Through the detection of signals, we have identified eight categories of drugs associated with vitiligo, among which antineoplastic drugs and immune inhibitors constitute the majority. Notably, these two drug categories showed a significant annual increase from 2004 to 2024 (*p*‐adjust < 0.05). Vitiligo is triggered by oxidative stress and autoimmune reactions that induce hyperactive interferon (IFN)‐γ signaling pathways and activate cytotoxic CD8+ T lymphocytes, causing melanocyte damage [19]. Therefore, drugs may induce vitiligo by activating T cells, increasing the release of inflammatory cytokines and antibodies related to immune‐related effects, or modulating immunoregulatory signaling pathways, such as those involving immune checkpoint inhibitors, cytokines, and their receptor antagonists. Enhanced pharmacovigilance systems and greater patient education initiatives may also contribute to the observed increase in adverse event reporting. These results highlight the critical need for rigorous surveillance and proactive management of medication‐associated complications, particularly for high‐risk drugs and patient populations.

Moreover, we noted that among the cases reported in the literature, disease severity prior to the onset of vitiligo induced by immune inhibitor treatment was predominantly classified as “severe” (12 out of 13 cases; 15 cases were excluded due to lack of severity description) [20, 21, 22, 23, 24, 25, 26, 27, 28, 29, 30, 31, 32, 33, 34, 35, 36, 37] (Table 4). The result suggested that disease severity might be predictive of the occurrence of immune inhibitor‐related vitiligo, which warrants verification in larger clinical cohorts.

**TABLE 4:** Previous reported cases of vitiligo associated with immune inhibitors to date.

Immune inhibitors	Author, year	Age (year)	Sex	Biologic drugs indication	Severity of disease	Areas affected	Onset (month)	Previous history of vitiligo	Outcome
Dupilumab	Takeoka S et al. (2021)	17	M	AD	Severe	Forehead	3	Yes	Non‐fatal
	Ren H et al. (2023)	18	M	AD	Severe	Cheeks neck	3	No	Non‐fatal
		50	F	AD	Severe	Right hairline Neck chest	2	No	Non‐fatal
		40	F	AD	Severe	Hands feet	8	No	Non‐fatal
		50	M	AD	Severe	Hands Legs feet	1	No	Non‐fatal
		18	F	AD	Severe	Pelvis left leg lower torso	6.25	Yes	Non‐fatal
		43	F	NP	Severe	Face bilateral breasts Chest Abdomen bilateral forearms proximal thighs	15	Yes	Non‐fatal
		52	M	NP	Severe	Forehead Glabella Nose cheeks	2	Yes	Non‐fatal
	Picone V et al. (2023)	79	M	AD	Not reported	Scalp Neck back of the hands	1	No	Non‐fatal
Ixekizumab	Su HJ et al. (2023)	72	F	Psoriasis	Not reported	Face	4	No	Non‐fatal
	Federico Pirro et al. (2019)	48	M	Psoriasis	Severe	Legs hands feet	3	No	Non‐fatal
	Marasca C et al. (2021)	53	M	Psoriasis	Severe	Face	3	No	Non‐fatal
	Eker H et al. (2022)	71	M	Psoriasis	Not reported	Cheeks perioral areas	2	No	Non‐fatal
	Pathmarajah P et al. (2022)	36	M	Psoriasis	Severe	Trunk limbs face	11	No	Non‐fatal
Secukinumab	Bouzid S et al. (2023)	30	M	Psoriasis	Not reported	Hands Palms back	9	No	Non‐fatal
	Nieto‐Benito LM et al. (2020)	39	M	Psoriasis	Generalized	Axillae Neck arms feet	24	No	Non‐fatal
	Nieto‐Benito LM et al. (2020)	20	F	Psoriasis	Not reported	Axillary	12	No	Non‐fatal
	Kim JC et al. (2023)	38	M	Psoriasis	Not reported	Legs	21	Yes	Non‐fatal
	Giordano D et al. (2021)	42	F	Psoriasis	Not reported	Upper limbs trunk	12	No	Non‐fatal
Ustekinumab	Gedikli OK et al. (2020)	33	M	PsA	Not reported	The dorsum of the fingers	4	No	Non‐fatal
Infliximab	Carvalho CL et al. (2014)	46	F	RA	Not reported	Left upper limb left hemithorax	2	No	Non‐fatal
	Ramírez‐HernándezM et al. (2005)	61	M	RA	Not reported	The dorsa of the hands	6	No	Non‐fatal
	Mattox AR et al. (2013)	60	M	Pityriasis Rubra Pilaris	Not reported	The dorsal fingers left wrist	28	No	Non‐fatal
	Ryu TH et al. (2017)	34	M	Ulcerative colitis	Not reported	The right mandibular auricular area	4	No	Non‐fatal
	Lu X et al. (2019)	39	M	Psoriasis	Not reported	Forehead	3	No	Non‐fatal
	Ismail WA et al. (2011)	51	M	Ulcerative colitis	Not reported	Face	1	No	Non‐fatal
	Luber RP et al. (2017)	83	M	Crohn disease	Not reported	Limbs trunk	8	No	Non‐fatal
Guselkumab	Martora F et al. (2023)	60	M	PsA	Severe	Face	4	No	Non‐fatal

Vitiligo has been identified as a common adverse reaction associated with imiquimod and chloroquine, while we observed a high signal strength for these two drugs. This observation aligns with the information provided in their product labels, thereby reinforcing the safety concerns regarding the clinical application of these medications. There have been case reports of adverse reactions associated with vitiligo for antineoplastic drugs mogamulizumab and ipilimumab [38], which showed high RORs in our study. Mogamulizumab, a monoclonal antibody targeting C‐C chemokine receptor 4, enhances the host immune response by depleting regulatory T cells (Tregs), thereby augmenting its therapeutic efficacy. Ipilimumab, a humanized antibody, specifically inhibits cytotoxic T‐lymphocyte‐associated antigen 4 (CTLA‐4), resulting in increased T‐cell activation and elevated secretion of IL2. This loss of tolerance and checkpoint blockade, while therapeutically beneficial, may lead to the appearance of vitiligo [37, 39]. Notably, the development of clinical depigmentation could potentially function as an indicator of positive therapeutic response in cancer treatment. Specifically, the emergence of vitiligo‐like hypopigmentation in melanoma patients may correlate with improved treatment outcomes [40]. Therefore, clinicians should comprehensively evaluate patient risk factors before using these medications and maintain surveillance for potential vitiligo‐related AEs.

In our study, the median time to onset of drug‐induced vitiligo was 86 days. A substantial proportion of patients (51.00%) developed this adverse event within the initial 90 days of treatment, suggesting that drug‐related vitiligo predominantly manifests early in the therapeutic course. This observation emphasizes the necessity for vigilant monitoring of patients during this early phase and highlights the significance of patient education regarding the initial symptoms of vitiligo to facilitate timely intervention. Nevertheless, it should be acknowledged that vitiligo may also emerge more than a year after the commencement of medication.

Confounding by indication represents an important consideration in evaluating immune‐modulating therapies associated with vitiligo. Underlying diseases, including melanoma, atopic dermatitis, and psoriasis, may independently increase susceptibility to vitiligo and may therefore contribute to the observed disproportionality signals. To address this concern, we performed indication‐related sensitivity analyses. After exclusion of these indication‐related reports, the majority of drug‐associated vitiligo signals remained detectable, suggesting that the observed associations were not entirely explained by the underlying diseases. However, the magnitude of some signals was attenuated, particularly among immune checkpoint inhibitors after exclusion of melanoma‐related cases, indicating that disease‐related immune mechanisms may partially contribute to the observed associations.

This study has several limitations. First, both FAERS and CAVR are spontaneous reporting systems that are inherently subject to underreporting, incomplete reporting, and misreporting, potentially introducing reporting bias. Second, the reports in these databases often lack comprehensive information such as comorbidities, concomitant medications, treatment duration, and therapeutic history, which may confound the observed associations. Third, disproportionality analysis can only reveal statistical associations between drugs and AEs, but cannot establish causality. Therefore, prospective studies are warranted to further validate the findings of this study.

## 5 Conclusion

This study comprehensively analyzed of drug‐induced vitiligo using FAERS and the CVAR database, revealing a consistent upward trend in reports over the past 2 decades. Notably, strong signals were detected for antineoplastic agents and immune inhibitors. These findings are vital for healthcare providers, researchers, and regulatory authorities, highlighting the critical need for continuous monitoring and reassessment of drug safety to safeguard patient health.

## Author Contributions

Y.L., Y.O., L.C., R.W., and X.S. performed the research. Y.L., Y.O., X.S., and J.Z. designed the research study. J.Z. and J.C. contributed essential reagents or tools. Y.L., Y.O., L.C., R.W., X.S., and J.Z. analyzed the data. Y.L., Y.O., L.C., R.W., X.S., and J.Z. wrote the paper. J.Z. and J.C. supervised the study and acquired funding.

## Funding

This work was supported by the Program for Youth Innovation in Future Medicine, Chongqing Medical University. The Chongqing talent plan project, cstc2024ycjhbgzxm0177. The Chongqing Science and Technology Commission, 2023NSCQ‐MSX0321. Outstanding Youth Foundation of Chongqing Province, CSTB2024YCJH‐KYXM0101.China Postdoctoral Science Foundation, 2024M763893.The Innovative Research Project for Doctoral Students of the First Clinical College of Chongqing Medical University, CYYY‐BSYJSKYCXXM202428.

## Ethics Statement

The authors have nothing to report.

## Consent

The authors have nothing to report.

## Conflicts of Interest

The authors declare no conflicts of interest.

## Supporting information


**Table S1:** Annual reporting cases in CAVR database. The annual number of adverse event reports related to vitiligo from 1990 to 2024 in CAVR database.
**Table S2:** Global reporting of drug‐related vitiligo events by country.
**Table S3:** Signal detection of the drugs related to drug‐induced vitiligo.
**Table S4:** Indication‐related sensitivity analyses for drug‐associated vitiligo signals.

## Data Availability

The all datasets used for analyses are publicly available at: FDA Adverse Event Reporting System (FAERS): https://fis.fda.gov/extensions/FPD%E2%80%90QDE%E2%80%90FAERS/FPD%E2%80%90QDE%E2%80%90FAERS.html. Canada Vigilance Adverse Reaction Online Database: https://www.canada.ca/en/health%E2%80%90canada/services/drugs%E2%80%90health%E2%80%90products/medeffect%E2%80%90canada/adverse%E2%80%90reaction%E2%80%90database.html.
