# Supplementary material for: Drug‐Induced Vitiligo: FDA Adverse Events Reporting System (FAERS) and Canada Vigilance Adverse Reaction (CAVR) Database Analysis and Literature Review
Source: J Cosmet Dermatol. 2026 Aug 24;25(9):e71149. doi: 10.1111/jocd.71149 (PMC13503976; doi:10.1111/jocd.71149)
Supplement: Supplementary file 1 [file JOCD-25-e71149-s001.docx]

**Supplement Table S1.** Annual reporting cases in CAVR database. The annual number of adverse event reports related to vitiligo from 1990 to 2024 in CAVR database.

| **DrugCategory** | **% in 2003 (n = 1)** | **% in 2004 (n = 1)** | **% in 2006 (n = 2)** | **% in 2017 (n = 5)** | **% in 2018 (n = 35)** | **% in 2019 (n = 18)** | **% in 2021 (n = 4)** | **% in 2022 (n = 3)** | **% in 2023 (n = 22)** | **% in 2024 (n = 26)** |
| --- | --- | --- | --- | --- | --- | --- | --- | --- | --- | --- |
| Antineoplastic drugs | 0 | 0 | 0 | 100 | 2.86 | 11.11 | 0 | 0 | 9.09 | 0 |
| Immune inhibitors | 0 | 0 | 0 | 0 | 91.43 | 77.78 | 0 | 0 | 90.91 | 84.62 |
| Immunomodulatory drugs | 100 | 100 | 100 | 0 | 0 | 0 | 0 | 0 | 0 | 0 |
| Others | 0 | 0 | 0 | 0 | 5.71 | 11.11 | 100 | 100 | 0 | 15.38 |

CAVR, Canada Vigilance Adverse Reaction.

**Supplement Table S2.** Global reporting of drug-related vitiligo events by country.

| **Reported Countries** | **Number of Cases** | **Percentage** |
| --- | --- | --- |
| United states | 983 | 51.47% |
| France | 140 | 7.33% |
| Germany | 84 | 4.40% |
| United kingdom | 84 | 4.40% |
| Canada | 62 | 3.25% |
| Brazil | 51 | 2.67% |
| Italy | 47 | 2.46% |
| Spain | 35 | 1.83% |
| Japan | 32 | 1.68% |
| China | 28 | 1.47% |
| Australia | 19 | 0.99% |
| Serbia | 17 | 0.89% |
| Poland | 14 | 0.73% |
| Sweden | 14 | 0.73% |
| Argentina | 13 | 0.68% |
| Bulgaria | 13 | 0.68% |
| Israel | 13 | 0.68% |
| Colombia | 12 | 0.63% |
| Turkey | 12 | 0.63% |
| Portugal | 11 | 0.58% |
| Switzerland | 10 | 0.52% |
| Czechia | 9 | 0.47% |
| Croatia | 9 | 0.47% |
| India | 9 | 0.47% |
| Netherlands | 8 | 0.42% |
| Greece | 7 | 0.37% |
| Norway | 7 | 0.37% |
| Panama | 7 | 0.37% |
| Austria | 6 | 0.31% |
| Costa rica | 6 | 0.31% |
| Mexico | 6 | 0.31% |
| Romania | 6 | 0.31% |
| Taiwan | 6 | 0.31% |
| Egypt | 5 | 0.26% |
| Nigeria | 5 | 0.26% |
| Puerto rico | 5 | 0.26% |
| Hungary | 4 | 0.21% |
| South korea | 4 | 0.21% |
| Slovenia | 4 | 0.21% |
| Cameroon | 3 | 0.16% |
| Singapore | 3 | 0.16% |
| South africa | 3 | 0.16% |
| United arab emirates | 2 | 0.10% |
| Algeria | 2 | 0.10% |
| Belgium | 2 | 0.10% |
| Botswana | 2 | 0.10% |
| Chile | 2 | 0.10% |
| Denmark | 2 | 0.10% |
| Ecuador | 2 | 0.10% |
| Finland | 2 | 0.10% |
| Iran | 2 | 0.10% |
| Morocco | 2 | 0.10% |
| New zealand | 2 | 0.10% |
| Saudi arabia | 2 | 0.10% |
| Dominican republic | 1 | 0.05% |
| Algeria | 1 | 0.05% |
| Hong kong | 1 | 0.05% |
| Ireland | 1 | 0.05% |
| Iran (islamic republic of) | 1 | 0.05% |
| Kazakhstan | 1 | 0.05% |
| Lebanon | 1 | 0.05% |
| Lithuania | 1 | 0.05% |
| Martinique | 1 | 0.05% |
| Philippines | 1 | 0.05% |
| Russia | 1 | 0.05% |
| Slovakia | 1 | 0.05% |
| Thailand | 1 | 0.05% |
| Tunisia | 1 | 0.05% |
| Trinidad and tobago | 1 | 0.05% |
| Venezuela | 1 | 0.05% |
| Vietnam | 1 | 0.05% |
| Unknown | 53 | 2.77% |

**Supplement Table S3.** Signal detection of the drugs related to drug-induced vitiligo.

| **Drug Category** | **Drug** | **Number of Cases** | **ROR** | **95%CI** |
| --- | --- | --- | --- | --- |
| **Fears** |  |  |  |  |
| Antineoplastic drugs | Nivolumab | 152 | 25.08 | 21.24 - 29.60 |
|  | Pembrolizumab | 132 | 28.73 | 24.07 - 34.30 |
|  | Ipilimumab | 67 | 38.97 | 30.52 - 49.75 |
|  | Ribociclib | 36 | 21.06 | 15.14 - 29.30 |
|  | Imatinib | 11 | 1.91 | 1.05 - 3.45 |
|  | Anastrozole | 8 | 5.39 | 2.69 - 10.80 |
|  | Dabrafenib | 8 | 6.06 | 3.03 - 12.14 |
|  | Abemaciclb | 6 | 4.80 | 2.15 - 10.69 |
|  | Fulvestrant | 6 | 5.69 | 2.55 - 12.68 |
|  | Trametnib | 6 | 6.12 | 2.74 - 13.64 |
|  | Tamoxifen | 5 | 9.51 | 3.95 - 22.88 |
|  | Vemurafenib | 4 | 4.12 | 1.54 - 10.99 |
|  | Encorafenib | 3 | 6.43 | 2.07 - 19.96 |
|  | Exemestane | 3 | 3.91 | 1.26 - 12.15 |
|  | Letrozole | 24 | 11.8 | 7.89 - 17.66 |
|  | Temzolomide | 10 | 5.84 | 3.14 - 10.87 |
|  | Pemetrexed | 7 | 5.39 | 2.56 - 11.32 |
|  | Busulfan | 4 | 6.31 | 2.37 - 16.84 |
|  | Fludarabine | 3 | 3.67 | 1.18 - 11.39 |
|  | Atezolizumab | 28 | 12.64 | 8.70 - 18.37 |
|  | Alemtuzumab | 19 | 16.61 | 10.56 - 26.10 |
|  | Mogamulizumab | 10 | 73.93 | 39.62 - 137.94 |
|  | Interferon α-2b | 5 | 15.80 | 6.56 - 38.03 |
|  | Talimogene laherparepvec | 4 | 29.63 | 11.09 - 79.16 |
|  | Aldesleukin | 3 | 46.47 | 14.94 - 144.62 |
|  | Pembrolizumab | 132 | 28.73 | 24.07 - 34.30 |
| Immune inhibitors | Dupilumab | 77 | 2.64 | 2.10 - 3.31 |
|  | Secukinumab | 49 | 3.76 | 2.83 - 5.00 |
|  | Usteknumab | 31 | 4.17 | 2.93 - 5.95 |
|  | Ixekizumab | 16 | 5.29 | 3.23 - 8.65 |
|  | Risankizumab | 15 | 2.86 | 1.72 - 4.76 |
|  | Omalizumab | 12 | 2.05 | 1.16 - 3.62 |
|  | Guselkumab | 9 | 3.89 | 2.02 - 7.50 |
| Antibacterial and antifungal drugs | Amoxicillin clavulanic acid | 9 | 5.74 | 2.98 - 11.04 |
|  | Terbinafine | 3 | 3.28 | 1.06 - 10.19 |
| Immunomodulatory drugs | Tofacitinib | 33 | 2.54 | 1.80 - 3.59 |
|  | Imiquimod | 15 | 72.00 | 43.24 - 119.89 |
| Anti-hypertensive drugs | Amlodipine | 10 | 1.96 | 1.06 - 3.66 |
|  | Valsartan | 8 | 3.17 | 1.58 - 6.35 |
|  | Bisoprolol | 7 | 6.23 | 2.97 - 13.09 |
|  | Diltiazem | 5 | 5.12 | 2.13 - 12.31 |
| Gastric acid secretion inhibitor | Esomeprazole | 14 | 1.97 | 1.17 - 3.34 |
|  | Omeprazple | 13 | 3.28 | 1.90 - 5.67 |
|  | Pantoprazole | 7 | 2.19 | 1.04 - 4.60 |
|  | Rabeprazole | 3 | 7.51 | 2.42 - 23.32 |
| Corticosteroids | Prednisolone | 8 | 2.34 | 1.17 - 4.68 |
|  | Betamethasone | 3 | 5.47 | 1.76 - 16.99 |
|  | Clobetasol | 3 | 6.50 | 2.09 - 20.17 |
| Antithyroid drugs | Teprotumab | 3 | 6.58 | 2.12 - 20.44 |
|  | Levothyroxine | 10 | 1.90 | 1.02 - 3.53 |
| Others | Finasteride | 15 | 11.39 | 6.85 - 18.94 |
|  | Lamivudine | 5 | 4.68 | 1.94 - 11.26 |
|  | Alendronic acid | 16 | 4.43 | 2.71 - 7.24 |
|  | Azelaic acid | 3 | 26.45 | 8.51 - 82.20 |
|  | Chloroqunie | 3 | 53.33 | 17.13 - 166.02 |
|  | Sulfasalazine | 5 | 6.48 | 2.69 - 15.60 |
|  | Methylphenidte | 23 | 5.82 | 3.86 - 8.79 |
| **CAVR database** |  |  |  |  |
| Antineoplastic drugs | Keytruda | 3 | 44.48 | 14.06 - 140.72 |
|  | Nivolumab | 3 | 23.90 | 7.56 - 75.60 |
|  | Ipilimumab | 3 | 28.09 | 8.88 - 88.85 |
| Immune inhibitors | Omalizumab | 7 | 2.19 | 1.01 - 4.74 |
| Immunomodulatory drugs | Imiquimod | 6 | 781.52 | 339.28 - 1800.21 |
| Others | Chloroquine | 3 | 317.07 | 99.92 - 1006.10 |
|  | Comirnaty | 6 | 11.08 | 4.84 - 25.38 |

CI, confidence interval; ROR, reporting odds ratio.

**Supplement Table S4.** Indication-related sensitivity analyses for drug-associated vitiligo signals.

| **Drug** | **Primary indication considered as confounder** | **Original ROR (95% CI)** | **Sensitivity ROR (95% CI)** |
| --- | --- | --- | --- |
| Nivolumab | Melanoma | 25.08 (21.24 - 29.6) | 9.2 (6.64-12.75) |
| Pembrolizumab | Melanoma | 28.73 (24.07 - 34.3) | 8.04 (2.01 - 32.17) |
| Atezolizumab | Melanoma | 12.64 (8.7 - 18.37) | 18 (12.47 - 25.99) |
| Dupilumab | Atopic dermatitis | 2.64 (2.1 - 3.31) | 3.17 (2.29 - 4.39) |
| Secukinumab | Psoriasis | 3.76 (2.83 - 5) | 2.47 (1.37 - 4.47) |
| Ixekizumab | Psoriasis | 5.29 (3.23 - 8.65) | 5.86 (2.2 - 15.64) |
| Risankizumab | Psoriasis | 2.86 (1.72 - 4.76) | 2.33 (0.75 - 7.22) |
| Guselkumab | Psoriasis | 3.89 (2.02 - 7.5) | 8.82 (3.96 - 19.67) |
| Ustekinumab | Psoriasis | 4.17 (2.93 - 5.95) | 2.04 (0.97 - 4.3) |

CI, confidence interval; ROR, reporting odds ratio.
